# NETs and CF Lung Disease: Current Status and Future Prospects

**DOI:** 10.3390/antibiotics4010062

**Published:** 2015-01-15

**Authors:** Robert D. Gray, Brian N. McCullagh, Paul B. McCray

**Affiliations:** Department of Pediatrics, Carver College of Medicine, University of Iowa, Iowa City, IA 52242, USA; E-Mails: r.d.gray@ed.ac.uk (R.D.G.); brian-mccullagh@uiowa.edu (B.N.M.)

**Keywords:** cystic fibrosis, Neutrophil, NETs

## Abstract

Cystic Fibrosis (CF) is the most common fatal monogenic disease among Caucasians. While CF affects multiple organ systems, the principle morbidity arises from progressive destruction of lung architecture due to chronic bacterial infection and inflammation. It is characterized by an innate immune defect that results in colonization of the airways with bacteria such as *Staphylococcus aureus* and *Pseudomonas aeruginosa* from an early age. Within the airway microenvironment the innate immune cells including epithelial cells, neutrophils, and macrophages have all been implicated in the host defense defect. The neutrophil, however, is the principal effector cell facilitating bacterial killing, but also participates in lung damage. This is evidenced by a disproportionately elevated neutrophil burden in the airways and increased neutrophil products capable of tissue degradation, such as neutrophil elastase. The CF airways also contain an abundance of nuclear material that may be originating from neutrophils. Neutrophil extracellular traps (NETs) are the product of a novel neutrophil death process that involves the expulsion of nuclear material embedded with histones, proteases, and antimicrobial proteins and peptides. NETs have been postulated to contribute to the bacterial killing capacity of neutrophils, however they also function as a source of proteases and other neutrophil products that may contribute to lung injury. Targeting nuclear material with inhaled DNase therapy improves lung function and reduces exacerbations in CF and some of these effects may be due to the degradation of NETs. We critically discuss the evidence for an antimicrobial function of NETs and their potential to cause lung damage and inflammation. We propose that CF animal models that recapitulate the human CF phenotype such as the *CFTR*^−/−^ pig may be useful in further elucidating a role for NETs.

## 1. Introduction

Cystic fibrosis is a multisystem disease caused by mutations in the cystic fibrosis transmembrane conductance regulator (*CFTR*) gene, which codes for a phosphorylation and nucleotide activated anion channel, resulting in altered transcellular chloride and bicarbonate transport. In excess of 85% of people with CF will die prematurely from respiratory complications, in spite of recent therapeutic advances [1]. The exact mechanisms leading to lung destruction and progressive respiratory failure have not been fully elucidated but are in part the result of a host defense defect that impairs bacterial killing, abnormal mucociliary transport, and a dysfunctional innate immune response to infection [2]. These mechanisms contribute to airway colonization with bacteria such as *Staphylococcus aureus* and *Haemophilus influenzae* early in life and with *Pseudomonas aeruginosa* through later childhood and adolescence [3].

## 2. Investigating Innate Immunity in CF Using Animal Models

The lung innate immune system includes airway epithelial cells, neutrophils, macrophages, and a vast array of proteins and peptides produced by these cells. Understanding how innate immunity is altered in CF is key to understanding the onset and progression of lung disease in early childhood. Progress has been hampered by the lack of an animal model that mirrors the lung disease features of people with CF. For example, *cftr*^−/−^ mice developed following the discovery of CFTR lack key features of lung disease seen in the human [4]. The advent of the CF pig has facilitated advancements in our understanding of CF lung disease as, like man, it develops a spontaneous lung disease phenotype with spontaneous bacterial colonization and associated innate immune dysfunction when CFTR function is lost [5,6]. CF pigs also display defective mucociliary transport and abnormal mucous properties [7,8], both features of early CF lung disease in humans. Furthermore, ferrets with loss of CFTR function also develop lung disease with similarities to the human disease [9].

## 3. Airways Surface Liquid (ASL) Antimicrobial Peptides in CF

ASL is a first line of defense against potential pathogens and contains secreted products of surface and submucosal gland epithelia. ASL is comprised of a mucous layer that traps inhaled or aspirated organisms and an aqueous layer in which cilia beat. ASL acts as a physical barrier to microorganisms but also contains numerous antimicrobial proteins and peptides (AMPs) to combat potentially harmful bacteria, fungi, and viruses [10]. These include lysozyme, lactoferrin, cathelicidins, β-defensins, secretory leukocyte protease inhibitor (SLPI), and the collectins surfactant proteins A and D (SP-A and SP-D). These multiple AMPs interact in an additive or synergistic fashion to inactivate pathogens [11]. ASL pH is in part regulated by HCO_3_^−^ secretion via CFTR; loss of CFTR function leads to acidification of ASL, a feature reported in primary cell culture of epithelial cells [12] and submucosal glands [13], as well as airways breath condensate from CF patients [14], and more recently in the CF pig [15]. Low (acidic) pH impairs the function of several antimicrobial peptides such as lysozyme, lactoferrin, cathelicidin LL37, SP-A and -D, and SLPI [10]. ASL acidification in the CF pig impairs bacterial killing at birth, and raising ASL pH in CF pig airways reverses this defect. Conversely, the antimicrobial activity of ASL of non-CF pigs was impaired by lowering the pH. *In vitro* studies with individual AMPs, including lactoferrin and lysozyme, demonstrated that the factors antibacterial properties were pH dependent. Together, these studies provide *in vivo* evidence of a primary bacterial killing defect due to impaired AMP function in a low pH environment [15].

## 4. Neutrophils and Antimicrobial Defense in CF

**Figure 1:**
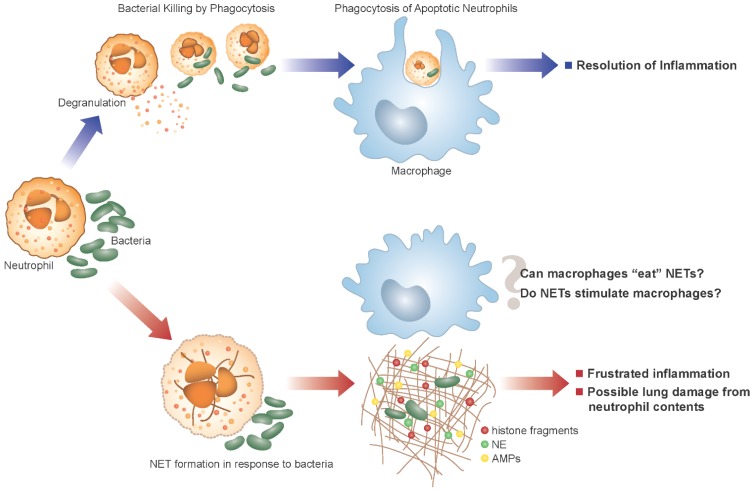
Alternative fates of Neutrophils in the Cystic Fibrosis (CF) lung. In the normal lung (purple arrows/top panel), neutrophils encounter and phagocytose bacteria. Following phagocytosis neutrophils rapidly undergo apoptosis and clearance by macrophages thus promoting resolution of inflammation. Alternatively, the excess of bacteria in the CF airway (red arrows/bottom panel) may lead to neutrophils forming neutrophil extracellular traps (NETs) in addition to normal phagocytosis. NETs may contribute to host defense, but also allow the release of toxic components into the airway that can damage the host lung. We can postulate that NETs contribute to the failed resolution of inflammation in the CF lung, and the clearance of NETs by macrophages may not be as anti-inflammatory as the clearance of apoptotic neutrophils.

ASL also contains resident immune cells that act as a first line of host defense against inhaled pathogens, with the neutrophil being of primary importance as a phagocyte. Neutrophils are recruited to the lungs as part of the innate immune response to microbes (Figure 1). The airways of infants and children with CF are characterized by a disproportionately elevated neutrophil burden either attributed to early microbial challenge [16,17,18] or, in the absence of microbes, to dysfunctional CFTR trafficking [19,20,21]. In spite of this response, bacteria such as *Staphylococcus aureus*, *Haemophilus influenzae*, and *Pseudomonas aeruginosa* persist in the airways and establish chronic infection. Neutrophils have granules that contain a wide array of products such as proteolytic enzymes, AMPs, myeloperoxidase (MPO), and peptides that degrade bacteria following phagocytosis. These products may also be released by degranulation to assist in the extracellular killing of organisms [22]. Phagocytosis is a primary function of neutrophils and is composed of three processes: (1) receptor mediated pathogen uptake into vacuoles (phagosomes); (2) production of reactive oxygen species in the vacuole (later utilized by MPO to form hypochlorous acid); and (3) the fusion of granules containing proteases and antimicrobial mediators to these vacuoles (the phagolysosome) resulting in destruction and digestion of pathogens [22]. There is evidence to suggest that microbicidal function of neutrophils is impaired in CF as a result of reduced chloride anion availability for the production of hypochlorous acid, an essential mediator in the destruction of pathogens in the phagolysosome [23]. Phagocytosis typically accelerates neutrophil apoptosis; in turn, macrophages recognize and phagocytose the aging neutrophil which ultimately promotes an orderly resolution of inflammation [24,25]. Neutrophils also release proteases and AMPs by degranulation with extracellular levels of these proteins increasing as the neutrophil burden increases in the lung [26]. AMPs produced by neutrophils include lysozyme, phospholipase A2, bacterial permeability increasing protein (BPI), lactoferrin, cathelicidins such as LL37, and the α-defensins or human neutrophil defense peptides 1–4 (HNP). In CF the proteolytic degradation of AMPs can further reduce their effectiveness and exacerbate local tissue damage [27,28].

Neutrophil proteases have the potential to damage the host if present extracellularly instead of targeted to the phagolysosome. Neutrophil elastase (NE), a serine protease contained in azurophilic (primary) granules, can degrade nearly all structural proteins of the lung [29], as well as reduce ciliary motility and facilitate bacterial colonization [16]. NE is present early on in the airways of infants and young children with CF, and serves as a predictor of disease progression and is associated with lung function decline, as reported in the ARREST CF study [30]. The quantity of NE released into CF airways overwhelms the anti-protease capacity, leading to detectable NE activity and local architectural destruction. Therefore, a paradox exists in CF whereby an abundance of resident antimicrobial material, including AMPs and proteases, has the potential to damage the host. Moreover, in spite of the abundance of antimicrobial products, chronic lung infection is the characteristic phenotype in people with CF.

## 5. Neutrophil Extracellular Traps and CF

Neutrophil extracellular trap (NET) formation was described in 2004 by Brinkmann *et al*. [31] and has rapidly gained prominence in the literature as an antibacterial defense mechanism employed by neutrophils to kill microorganisms (by a mechanism distinct from phagocytosis or degranulation). Now termed NETosis, the process of NET formation is characterized by neutrophils undergoing an oxidative burst and releasing webs of decondensed DNA complexed to peptides and proteins, accompanied by cell death [32]. NETs have been described in CF sputum by a number of investigators [33,34,35,36], and recently it has been suggested that the majority of extracellular DNA in the CF lung is derived from NETs [33]. Oxidative burst and NADPH oxidase activation are thought to be central mechanisms in NET formation [32] and both MPO and NE are deemed essential co-factors [37,38]. The oxidative burst ultimately leads to downstream activation of peptidyl dearginase 4 (PAD4) which translocates to the nucleus and modifies histones (H3 and H4) by (hyper)citrinulation which contributes to nuclear decondensation and NET formation [39]. This is supported by evidence that neutrophils from PAD4^−/−^ mice do not make NETs, and furthermore PAD4^−/−^ mice are more susceptible to challenge with bacteria [40]. Neutrophil elastase complexes are involved in the decondensation of chromatin and consequently NET formation can be inhibited by neutrophil elastase inhibitors [38]. Furthermore, recent evidence suggests that MPO is an important co-factor in elastase release from granules and its proteolytic activity during NET formation [41]. Additional evidence implicates autophagic activity in NETosis as an essential partner to the oxidative burst demonstrating that inhibition of either system stopped NETosis. Indeed the promotion and inhbition of autophagy have now been demonstrated to alter NET formation in a number of studies [42,43]. The normal fate of a neutrophil is to perform its role in host defense and die by apoptosis. Why neutrophils (responding to infection) may favor a radically different form of cell death than apoptosis is intriguing and an additional hypothesis proposes that CF neutrophils do not undergo apoptosis or NET formation but rather switch to an alternate “hyperexocytosing” phenotype and release their primary granule content including MPO and NE into the airway lumen by enhanced exocytosis [44,45,46]. Therefore, further investigation of the process and relevance of NET formation in CF is required.

Initial studies demonstrated NE, MPO, and histones as the main proteins associated with NETs [31,32,47]. Further proteomic studies have revealed a major presence of AMPs on NETs with lysozyme, lactoferrin, HNPs, and calprotectin all demonstrated as NET constituents [48,49], adding to the hypothesis that NETs play a role in bacterial killing. Histones are also believed to have a major role as an antimicrobial on NETs [47]. Cathelicidins, although not identified in proteomic studies of NET formation have nevertheless been described as NET proteins [50,51]. Therefore, NETs could be a rich source of AMPs in the CF airway, but the functional significance of this has yet to be elucidated, particularly in the context of chronic infection.

## 6. NET Formation and Killing in Response to Bacteria

A number of groups have demonstrated the ability of *Pseudomonas aeruginosa* to induce NET formation, with reference to CF lung disease [33,36,52,53,54]. Interestingly, two groups independently demonstrated that NET induction using clinical isolates of *Pseudomonas* results in a divergent response, namely isolates from early disease induce NETs but isolates from the same patients in later disease do not [36,53]. Furthermore pyocyanin, a bacterial toxin produced by *Pseudomonas* can induce NET formation [55] but can also induce neutrophil apoptosis [56]. These observations require further investigation to help us understand the temporal relationship between *Pseudomonas* and NET production and why certain toxin challenges such as pyocyanin can result in disparate outcomes for neutrophils.

The ability of NETs to kill bacteria following their induction remains controversial. Early reports demonstrated that neutrophils, pre-activated with cytokines or phorbol ester to form NETs, could subsequently trap and kill pathogens in a manner independent of phagocytosis [31,57]. Young and colleagues demonstrated that killing of *Pseudomonas* did occur, even without the pre-activation of neutrophils. However, this NET-mediated killing only accounted for a small proportion of total bacterial killing compared to phagocytosis, unless the experiments were performed with neutrophils in suspension; the effect was lost if neutrophils were cultured under standard conditions [36]. Additionally, they demonstrated that the ability to kill *Pseudomonas* was lost when patient isolates from later stages in disease were used, suggesting this pathogen develops resistance to NET-mediated killing as CF lung disease advances. This finding was further reinforced by Dwyer and colleagues who demonstrated a decreased ability of NETs to kill mucoid strains of *Pseudomona*s [33]. Other reports suggest that NETs may not be as potent in killing as initially suggested. Mengazzi et al reported that NETs trap rather than kill microbes, with the majority of NET bound bacteria being live when liberated by DNase treatment [58], which is in keeping with a previous observation that NETs alone were not sufficient to kill bacteria and needed the addition of exogenous hydrogen peroxide to be truly bacteriocidal [59]. Nevertheless the interaction of *Pseudomonas* and NETs is an area of great interest in CF and whether this process is central to the development of lung disease or indeed involved the pathoadaptation of *Pseudomonas* in the CF airway will require further investigation as reviewed elsewhere recently [60].

To date, the majority of studies linking NETs to CF have concentrated on the interaction of neutrophils with *Pseudomonas*, however the ability of *S. aureus* to induce NET formation has been studied and confirmed [52,61,62,63]. *S. aureus* is a major pathogen in early CF lung disease, with colonization often pre-dating *Pseudomonas* acquisition by years. Furthermore, the ability of *S. aureus* to degrade NETs and escape specific protease activity has been described [61,64]. This could be significant in early CF lung disease if indeed killing of S. *aureus* by NETs was inefficient. Thus, further work utilizing CF isolates of *S. aureus* to induce NETs and assess killing capacity are required, particularly in models of early disease.

## 7. Potential Damaging Consequences of NETs in CF

CF sputum contains large amounts of NETs when measured by biochemical assays, and these are decorated with antimicrobial peptides, neutrophil proteins, and proteases [33]. Therefore, it is somewhat surprising that (in spite of these structures) chronic infection persists. This observation suggests that the innate antimicrobial properties of NETs may be over emphasized in the context of CF lung disease. The presence of large amounts of DNA in the CF airways and its potential contribution to CF pathogenesis was first acknowledged in the 1950s [65]. However, it was not until several decades later that targeting DNA degradation as a therapy was proved to be efficacious in reducing sputum viscosity, improving pulmonary function, and reducing the number of pulmonary exacerbations [66]. These data suggest that clearing NETs from the airway in CF is beneficial and this may account for some of the effects of DNase therapy. As previously stated NE (a key NET-associated protein) is present in high concentrations in CF, contributing to a protease/anti-protease imbalance in the lung. Cathepsin G and proteinase 3 are also released in NETs and likely add to the protease excess in the CF lung and damage tissue [52]. These data suggest that NETs may also represent a potentially deleterious source of proteases in the CF lung.

In addition to possibly contributing to protease-induced lung damage in CF, NETs may also be an important pro-inflammatory stimulus in the CF lung. NETs are a feature of autoimmune diseases characterized by significant levels of inflammation, and are implicated in anti-neutrophil cyoplasmic antibody (ANCA) vasculitis and associated kidney disease [67], and the pathogenesis of systemic lupus erythematosis (SLE). An impairment of DNAse degradation of NETs has been linked with the development of lupus nephritis [68], and further studies have demonstrated this inability to degrade NETs as more prominent in patients with other severe complications such as alopecia [69]. Un-degraded NET fragments activate complement as a central disease mechanism [70] and NETs in association with LL37 have been described as a source of extracellular antigen (acting via TLR-9) in SLE, which may also drive the disease process [71]. The presence of the cathelicidin on NETs has also been shown to promote atherosclerosis in mice by activating plasmacytoid dendritic cells [50] further suggesting that AMPs in association with DNA may promote inflammation. This feature may be highly relevant to CF lung disease, particularly when we consider that DNase is already employed as a successful therapy in CF.

## 8. Outstanding Questions in CF

Despite the presence of NETs (and associated AMPs, proteases, and other key neutrophil proteins) in the CF airway, there is unremitting bacterial infection. Thus we can infer that either NETs are inefficient in killing bacteria in CF lungs (possibly because the bacteria have evolved mechanisms to evade NET-based killing) or that NETs are simply trapping bugs rather than killing them. In fact, the process of trapping bacteria could be an important factor in stopping bacterial dissemination in CF and may be similar to extracellular trap encapsulation, a process seen in invertebrates, which possess only an innate immune system [72]. A further consideration is that phagocytosis and NET-mediated killing are overwhelmed by the volume of bacteria in the CF lung and simply attempt to hold infection in check. This is an attractive theory, particularly if we consider that low ASL pH, poor mucociliary transport, and other features of the environment of the CF lung could dampen the antimicrobial functions of NET-bound AMPs. Therefore, NETs in the CF lung may predominantly act as a source of damaging neutrophil products while being relatively impotent in antimicrobial activity. A further, as yet unanswered question, is whether the abundance of NETs in the CF lung is related to overproduction by CFTR-deficient neutrophils or a lack of clearance due to the reduced mucociliary transport in CF. Finally, there is a paucity of data on the mechanism of clearance of NETs with some evidence suggesting that NETs can be taken up by macrophages and degraded in the lysosome in an immunologically silent process [73]. However, further work is required to determine the mechanism for macrophage mediated clearance of NETs and how it affects the resolution of inflammation, which is normally driven by the efferocytosis of apoptotic neutrophils [25]. We propose that many of these questions could be answered by carefully designed experiments in appropriate systems and animal models such as the CF pig.

## 9. NETs Research in the CF Pig Model

**Figure 2:**
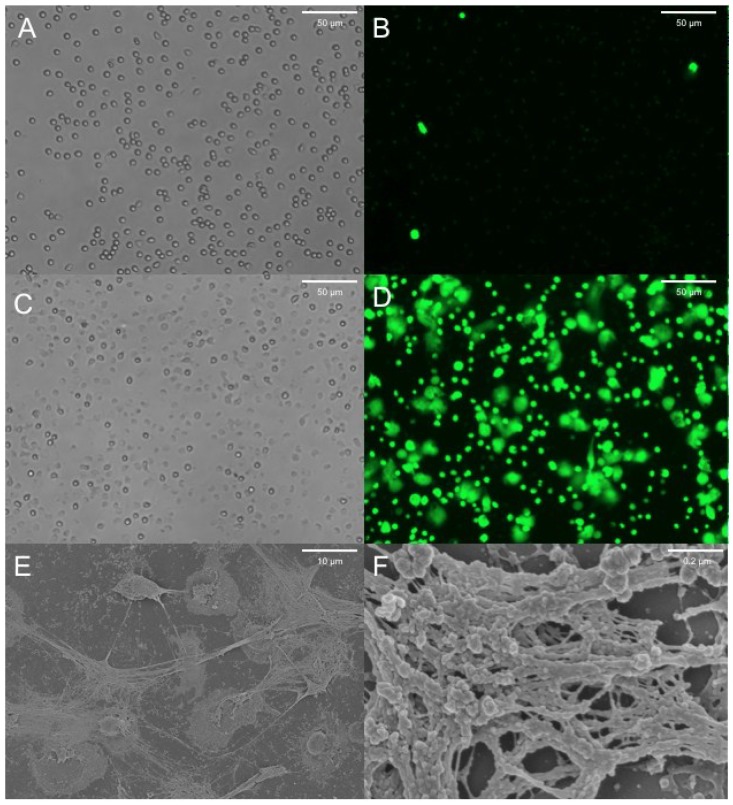
Porcine *CFTR*^−/−^ neutrophils stimulated with LPS to produce NETs. Cells were plated at a density of 50,000 cells per well in 24 well plates and treated for 6–8 h with LPS 100 μg/mL or control. Cells were then stained with Sytox green, which preferentially stains extracellular DNA and is excluded from live cells. (**A**) Brightfield image of untreated neutrophils showing normal morphology; (**B**) Sytox staining of untreated neutrophils demonstrates minimal staining for extracellular DNA; (**C**) Brightfield image of LPS treated neutrophils showing flattened and activated cells; (**D**) Sytox staining of LPS treated neutrophils showing characteristic NET structures; (**E**,**F**) Scanning electron microscopy (SEM) of fixed preparations at low and high magnification demonstrating characteristic mesh-like structures of NETs following LPS treatment.

The CF pig, like the human, develops significant gut and lung disease, the latter being characterized by neutrophil infiltration and lung destruction [6]. Human CF blood neutrophils demonstrate no impairment in releasing NETs when compared to non-CF neutrophils [36]. NET formation and the associated release of proteases have been demonstrated in wild type porcine neutrophils following calcium ionophore treatment [74]. We can demonstrate NET formation by neutrophils from *CFTR*^−/−^ animals following lipopolysaccharide (LPS) treatment (Figure 2). These porcine NETs show characteristic extracellular DNA release in *ex vivo* cell culture and associated features of NETosis by scanning electron microscopy. Thus, we can study the process of NETosis in neutrophils from a relevant large animal model of CF and potentially address many of the questions posed above. Furthermore by monitoring the development of lung disease in the CF pig model it may be possible to delineate the role of NET formation in this process. By measuring NET abundance in this model at different time points both longitudinally and following challenge with pathogens, we will be able to assess the contribution of NETs to the early pathogenesis of CF lung disease. Furthermore, therapeutic approaches to modulate NET production may be studied in an animal where repeated sampling and biopsy of the airway with standard techniques is possible.

## 10. Conclusions

NET formation is a potentially important mechanism in the development of CF lung disease with results to date showing that it likely occurs in response to bacterial infection. The evidence that NETs are a major antimicrobial mechanism in CF is at present incomplete. Further work is required to determine whether NETs in CF contribute to host defense (which is knowingly impaired in CF) or whether their main contribution is to inflammation and lung damage.
